# Molecular docking based screening of triterpenoids as potential G-quadruplex stabilizing ligands with anti-cancer activity

**DOI:** 10.6026/97320630013284

**Published:** 2017-09-30

**Authors:** Sittichai Sillapapongwarakorn, Somchai Yanarojana, Darawan Pinthong, Amnuay Thithapandha, Jiraporn Ungwitayatorn, Porntip Supavilai

**Affiliations:** 1 Department of Pharmacology, Faculty of Science, Mahidol University, 272 Rama 6 Road, Bangkok 10400, Thailand;; 2 Department of Pharmaceutical Chemistry, Faculty of Pharmacy, Mahidol University, 447 Sri-Ayudhaya Road, Bangkok 10400, Thailand;

**Keywords:** Ganoderma lucidum, G-quadruplex, molecular docking, triterpenoids, virtual screening

## Abstract

Triterpenoids isolated from Ganoderma lucidum (GLTs) exhibit a broad spectrum of anti-cancer properties, including anti-proliferative, anti-metastatic and anti-angiogenic activities. Current research studies revealed the role by GLTs in inducing apoptosis and suppression of telomerase activity of cancer cells with much lower toxicity to healthy cells. Compounds selectively binding and stabilizing G-quadruplex structures could inhibit the telomerase or downregulate the oncogenes and may act as anti-cancer agents. Targeting human telomeric G-quadruplex DNA could be one of the mechanisms by which these GLTs exert anti-cancer activity. In this study, 208 GLTs were screened for ligands with high binding affinity and selectively to stabilize the pG4DNA by using the docking tool AutoDock4. The results showed that ganoderic acid A and ganoderic acid Df exhibit high binding affinity and selectively bind to the lateral groove of pG4DNA. Based on our findings, we suggest that the triterpenoid represents a new class of G-quadruplex groove binding ligands and thus act as potential anti-cancer agents.

## Background

Ganoderma lucidum (GL) possesses various pharmacological properties, which are also documented in the ancient reports where GL is praised for its effects on the promotion of health and longevity. It has been known to have numerous pharmacological effects including anti-aging, anti-cancer, anti-diabetic, anti-HIV-1, anti-inflammatory, anti-hypertensive, anti-oxidative, immunemodulating, and radical-scavenging effects [1]. Recently, more than 400 bioactive compounds have been isolated and identified from GL [2]. The main bioactive natural components from GL are triterpenoids, polysaccharides, nucleotides, fatty acids, glycoproteins, sterols, steroids, proteins and trace elements [3]. GL is one attractive source of anti-cancer products, which have been used for centuries as an herbal medicine for the prevention and treatment of cancer and improving immune function. The triterpenoids, structurally highly oxidized lanostanes, have been isolated and characterized with ganoderic acids (GAs) such as ganoderic acid A (GA A), GA D, GA Df, GA T [3, 4]. Accumulated data have shown that GAs exhibits a broad spectrum of anticancer properties, including anti-proliferative, anti-metastatic and anti-angiogenic activities [5]. In 2008, Yuen et al. reported that GL extract (GLE) is a potential source of chemopreventive agents for human bladder cancer [6]. Cancer cell growth inhibition induced by GLE is mediated via apoptosis associated with suppression of telomerase activity and oxidative DNA damage [6]. Telomeric DNA contains repetitive DNA sequence (TTAGGG) n forming G-quadruplex (G4) structures; this structure inhibits telomerase activity that is required to maintain telomeres [7]. Therefore, the compounds those selectively binds to and stabilize G4 complex structures could inhibit the telomerase and suppress the gene transcription of oncogenes, which will result in senescence and apoptosis of cancer cells [8].

Several research groups have focused on the structure-based design approaches to develop the potential anti-cancer ligands with the capability to stabilize G4 [9]. Most G4 ligands, such as BRACO19, PIPER, quercetin, RHPS4, telomestatin and TmTyP4, are planar molecules, which comprise a planar p-rich rings structure, allowing them to intercalate into G4 and form a stable conformation [10, 11, 12, 13, 14, 15]. Recently, non-planar G4 ligands such as berberine, peimine, peiminine and steroid FG, could stabilize G4 complex through interaction in the lateral groove [16, 17, 18, 19]. GLTs are non-planar molecules, structurally similar to steroid FG, which may stabilize G4 [17, 18]. GA A was the first GA isolated from GL in 1982 and it shows no cytotoxicity to normal and cancer cells [20, 21]. GA A displays anti-cancer effects, such as anti-invasion, inhibition of NF-kB AP1/uPA, anti-proliferation, inhibition of JAK-STAT3, inhibition of farnesyl protein transferase [22, 23, 24, 25]. In this study, GA A was selected to elucidate the capability of GA A to stabilize G4. The parallel stranded DNA quadruplex d-(TTAGGGT) 4 (PDB code 1NP9) were selected for exploiting the interaction of GA A and G4 structure [26]. The aims of the present work were to search for novel GLT ligands with high binding affinity and selectivity for the pG4DNA, which may lead to the discovery of novel natural molecules as lead, compounds having potential anti-cancer activity.

## Methodology

### Preparation of ligand

The two-dimensional (2D) structures of a total of 208 triterpenoids isolated from Ganoderma lucidum were downloaded from the SCiFinder database. The 3D structures were created with SYBYL 8.0 of NECTEC server. Energy minimization was performed to find the optimum structure with lowest energy. Energy minimization of each structure was achieved by using standard Tripos force field (Powell method and 0.0001 kcal/mole energy gradient convergence criteria). Electrostatic charge was assigned by Gasteiger-Huckel, and iterations number was set to 3,000 rounds.

### Molecular docking

The structure of the parallel G-quadruplex d-(TTAGGGT)4 containing the human telomeric repeat was retrieved from the protein data bank (PDB code 1NP9); http://www.rcsb.org/pdb) (Gavathiotis and Searle 2003). Dockings of GLTs to unbound pG4DNA were carried out using AutoDock 4 with AutoDockTools 1.5.6rc3 (ADT) as described by Li J et al. [18]. G4 structures were prepared for docking using Sybyl 8 software (Certara Inc. Princeton, USA) and ADT. The Gasteiger atomic charges and Kollman united atom partial charges were assigned for the GLTs and G4, respectively. Grid maps were set at four grooves and two terminal places for G4 and the grid boxes were centered at G4. The size of grid box was 60 x 60 x 60 in three dimensions. The grid was set to be sufficiently large to cover significant portions of the active sites. Lamarckian genetic search algorithm was employed. Maximum number of energy evaluation was 2, 500, 000 per run and population size was set at 150. All other parameters were set to default values. Two hundred fifty independent docking runs were performed. Results were divided into groups using the clustering module in ADT according to the 2.0 root-mean-square deviation (RMSD) criteria. Besides RMSD clustering, the binding free energies were evaluated for the binding conformations of ligand by using ADT, and the low-energy conformations were selected from the largest cluster [18].

### Molecular dynamics

Molecular dynamic (MD) simulations for the GLTs-G4 systems were carried out with the sander module of AMBER 12.0 program package as described by Li J et al. [18]. The PARM 99 parameters and General Amber Force Field (GAFF) parameters were set for G4 and GLTs, respectively. Partial-atomic charges for the triterpenoids were derived using Gaussian 03 with the HF/6- 31G (d) basis set followed by RESP calculation. Internal K+ ion was added into the channels of the cavities between consecutive guanine tetrads. Then, the TIP3P water model was chosen and extended to a distance of 10 Å from any solute atom. Counter K+ ion neutralized the systems.

Firstly, a 1000-step minimization was carried out with the solute molecules fixed, and the equilibration was continued by 20 ps of PME dynamics with the same restriction. Subsequently, five rounds of 1,000-step minimization followed this equilibration with solute restraints from 20 to 0 kcal/mol Å-2 reduced by 5 kcal kcal/mol Å-2 in the course of each round. Then, the system was heated from 0 to 300 K with the rate of 50 K for every 5 ps of MD run, and another 100 ps MD simulation was continued to equilibrate the system. After the minimization and equilibration, MD simulations were run under NPT condition at 300 K. During the MD simulations, SHAKE was used for constraining hydrogen atoms and a 9 cutoff was applied to non-bonded interaction. Simulation time step was set at 2 fs and the translational center of mass motion was removed every 10 ps [18].

## Results and Discussion

### Molecular docking and MD stimulations

**Figure 1:**
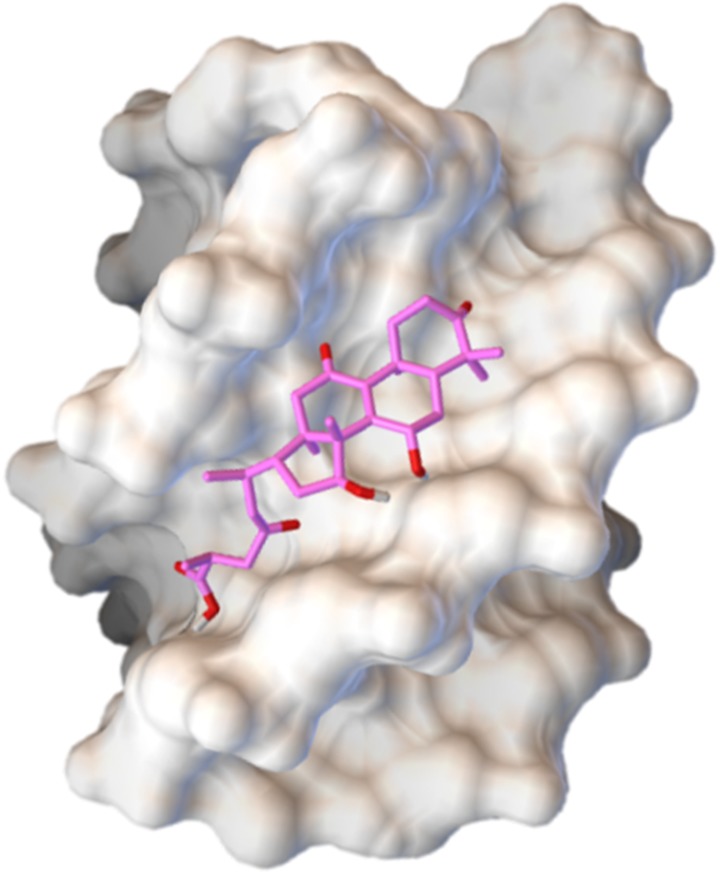
Predicted binding mode and the possible binding site of GA A with pG4DNA. GA A, represented in pink sticks, was able to enter and filled the binding groove of pG4DNA. Abbreviations: pG4DNA, parallel G-quadruplex DNA; GA, ganoderic acid.

GA A, which exhibits anti-cancer effects and shows no cytotoxicity to normal and cancer cells [20, 21], was selected to evaluate the ability to stabilize pG4DNA. The docking result of GA A in pG4DNA binding site is shown in Figure 1. The lowest energy docked conformation of the most populated cluster (the largest cluster) was selected and taken into account for study the binding against pG4DNA. The estimated inhibition constant (Ki) and estimated free binding energy (rG) of GA A which stabilized the pG4DNA are shown in Table 1. The lowest binding free energy conformation of GA A binding in pG4DNA was selected for further MD stimulation.

**Figure 2:**
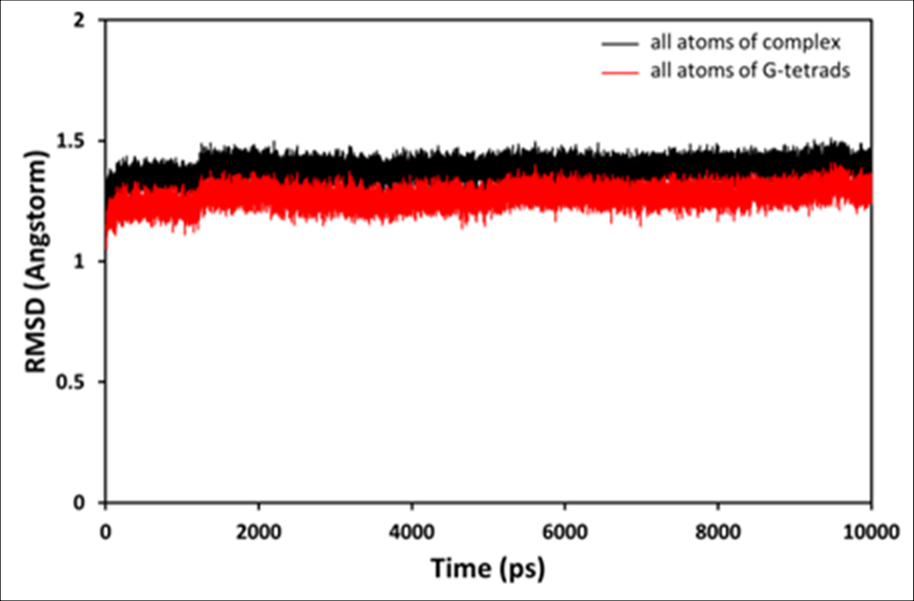
Structural drift observed during the MD simulation. RMSD plot showing the stability of the model during the MD run. RMSD values calculated for all atoms of GA A-pG4DNA complex (red) and backbone only atoms of pG4DNA (black) were plotted.

**Figure 3:**
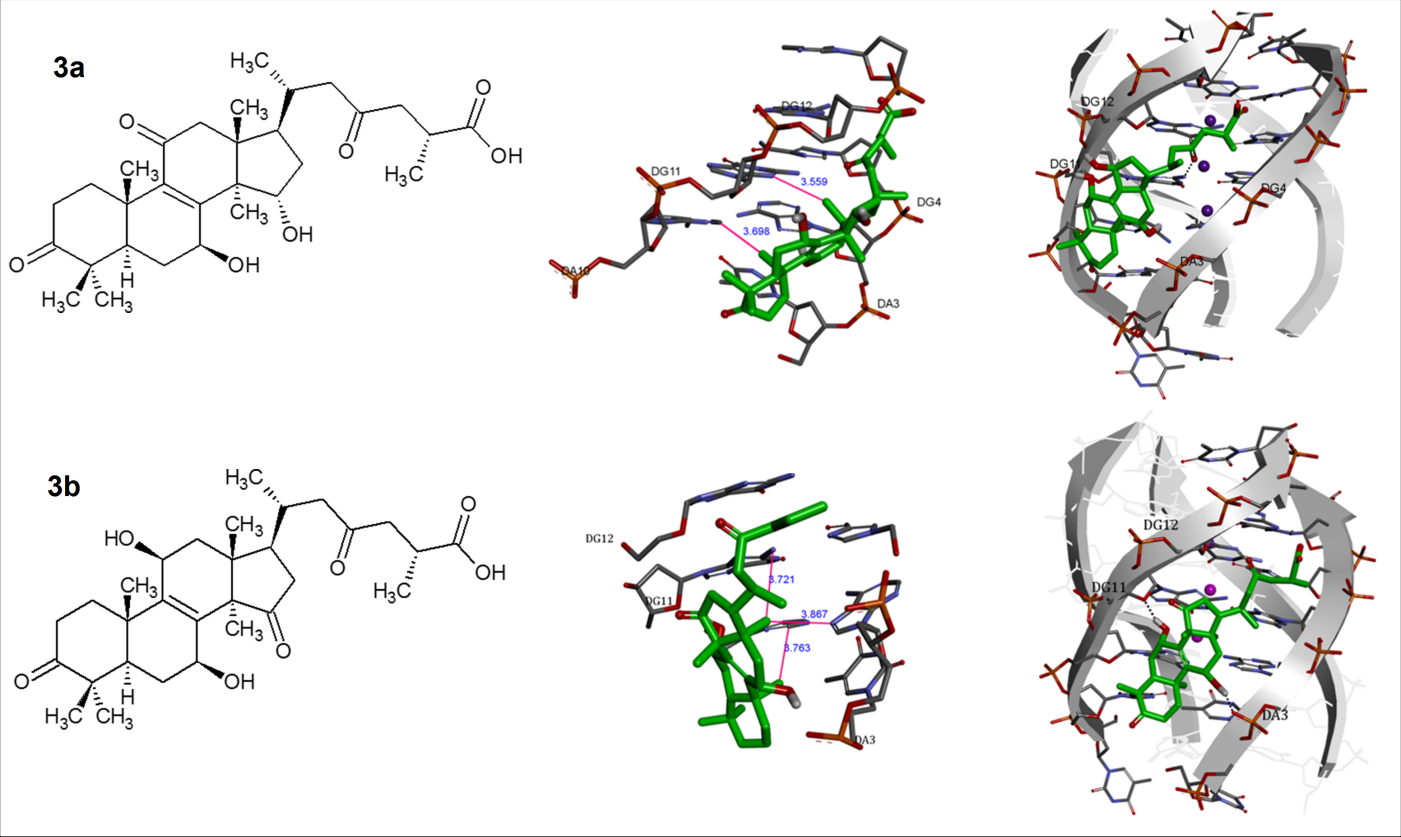
Binding poses of the (a) GA (Ganoderic acid) A and (b) GA (Ganoderic acid) Df in the pG4DNA-binding site. The figures shown are the predicted interactions formed by the ganoderic acid A in the active site. The compounds are represented in green sticks. The purple balls are K+. The pG4DNA structure is shown as a gray ribbon diagram with exception to the activation loop containing the DA-motif and DG-motif, which is shown in red sticks. The black dash lines represent hydrogen bonds, and purple lines denote hydrophobic interactions. Abbreviations: DA3, adenine base position 3 of G4DNA; DG11, guanine base position 11 of pG4DNA; pG4DNA, parallel G-quadruplex DNA; GA, ganoderic acid.

Measuring the RMSD over the course of the MD simulation assessed the conformational stability of the GA A-pG4DNA complex. The overall RMSD for all atoms of GA A-pG4DNA complex (red) and backbone-only atoms of pG4DNA (black) are illustrated in Figure 2. There were very few differences in the RMSD values observed between an all atom of GA A-pG4DNA complex and backbone-only model for the G-quartets. The stability of GA A-pG4DNA complex using RSMD calculations revealed that the binding of GA A was stable. Figure 3 (a)depicts GA A binding in the groove of pG4DNA through hydrogen bond and van der Waals interactions. Thus, GA A could be a potential novel natural molecule that can stabilize pG4DNA. Recently, more than 200 GLTs have been isolated and identified from GL [2]. Therefore, in silico screening of GLTs was performed to search for the GLTs with high binding affinity and selectivity for the pG4DNA.

### Virtual screening of GLTs as potential G4 stabilizing ligands

In this study, 208 triterpenoids isolated from GL were screened for the ligand with high binding affinity and selectivity for the pG4DNA. GLTs were docked to the pG4DNA. The structures, estimated Ki and estimated rG of GLTs, which stabilized the pG4DNA, are shown in Table 1. The results indicated that 131 GLTs interact with the pG4DNA with high affinity (Ki < 1 mM). GA Df was the most potent GAs to stabilize the pG4DNA with Ki = 13.97 nM.

### MD stimulations

MD stimulations were performed on GA A and GA Df with pG4DNA to explore the binding poses in depth. Molecular Mechanics and Generalized Born Surface Area (MM/GBSA) were determined for the best ranking conformation molecule on the solvation forces involved in the stabilization of GA-pG4DNA complex. The estimated rG, estimated Ki and target residues involved in the hydrogen bonding of the best-docked poses are given in Table 2. The pG4DNA (1NP9: containing the human telomeric repeat) consists of four equivalent grooves [26]. The results showed that GA A interacts with pG4DNA in the groove through hydrogen bond and van der Waals interactions. One hydrogen bond was formed by side chain carbonyl group of GA A and guanine base position 11 of lateral groove of pG4DNA (DG11) with hydrogen bond length of 2.21 Å. Two methyl groups (C18 and C19) were pointed into the groove and bound with guanine bases by hydrophobic and van der Waals interactions. The distance between methyl group (C18) and carbon atom of DG11 was 3.7 Å, and the distance between methyl group (C19) and nitrogen atom of DA10 was 3.6 Å (Figure 3a and Table 2). GA Df stabilized pG4DNA with 3 van der Waals interactions and 2 hydrogen bonds with pG4DNA at DG 11 and adenine base position 3 (DA3) with hydrogen bond length of 2.12 Å and 2.74 Å, respectively (Figure 3b and Table 2). Hydroxyl group of ring B of GA Df formed hydrogen bond with DG 11 and hydroxyl group of ring C formed H-bond with DA 3. Two methyl groups (C18 and C19) were pointed into the groove and bound with guanine base by hydrophobic and van der Waals interactions. The distance between methyl group (C18) and nitrogen atom of DG11 was 3.7 Å and the distance of this methyl group and nitrogen atom of DA3 was 3.9 Å. The distance between methyl group (C19) and carbon atom of DA10 was 3.8 Å. The result also provided new insight into the design of G4 groove-targeted agents.

Furthermore, Table 2 shows the MM/GBSA binding energy calculation of GA A and GA Df to pG4DNA. GA A displayed a lower total binding energy (-23.46 ± 1.70 kcal/mol) than GA Df (- 13.32 ± 2.21 kcal/mol). However, in docking experiment, GA Df was about 30 times more active than GA A (Table 1). As for the results from MD, the interaction of GAs with pG4DNA was in solution which mimicked the physiological condition, the total binding energy of GA A was approximately 2 times better than GA Df. Further studies are required for clarify these results.

The obtained results are in agreement with the published nonplanar G4 ligands that GLTs stabilized G4 through the groove binding [16, 17, 18][19]. The GLTs interacted with the pG4DNA and enhanced G4 stabilization through hydrogen bonds and van der Waals interactions. At physiological condition GA A, noncytotoxic GLTS [20, 21], might be potential lead compounds for the development of new telomerase inhibitors. Thus, GA A may serve as the starting point for the design of a new class of highly selective groove binding of pG4DNA with anti-cancer effect.

## Conclusion

In conclusion, the first virtual screening of GLTs as potential G4 stabilizing ligands was presented. Binding poses and binding energies for GLT-pG4DNA complexes were calculated using molecular docking and molecular dynamics. The results indicated that GLTs significantly stabilized the pG4DNA through interaction with the lateral groove of G4 by hydrogen bonds and van der Waals forces. GA A and GA Df exhibit high binding affinity and selectivity for lateral groove of pG4DNA with theoretical binding efficiency in nanomolar range. The triterpenoid represents a new class of highly selective groove-binding molecules. Thus, GLTs exert their novel anti-cancer mechanism by stabilizing the pG4DNA through the groove binding.

## Competing interests

The authors declare that they have no conflict of interests.

## Figures and Tables

**Table 1:** Docking summary of pG4DNA (1NP9) with 208 currently known GLTs.

Compound No.	Compounds	CAS No.	Estimate rG (kcal/mol)	Estimate average Ki (nM)
1	GA A	81907-62-2	-8.76	376.92
2	GA AM1	149507-55-1	-9.79	66.95
3	GA AP	120462-50-2	-9.44	121.01
4	GA AP2	1082416-00-9	-8.09	1,170
5	GA AP3	1082416-03-2	-9.57	97.06
6	GA B	81907-61-1	-8.46	633.32
7	GA B8	105817-07-0	-9.34	141.46
8	GA B9	-	-8.95	274.61
9	GA C2	103773-62-2	-8.93	282.57
10	GA C5	673460-24-7	-9.41	126.81
11	GA C6	105742-76-5	-7.51	3,130
12	GA D	108340-60-9	-8.89	305.82
13	GA D1	-	-9	254.25
14	GA D2	97653-94-6	-9.23	171.1
15	GA Df	1352033-73-8	-10.72	13.97
16	GA DM	173075-45-1	-9	254.56
17	GA E	98665-14-6	-9.96	50.39
18	GA F	98665-15-7	-9.07	226.16
19	GA G	98665-22-6	-8.54	545.63
20	GA GS-1	1206781-64-7	-8.67	444.3
21	GA GS-2	1206781-65-8	-8.76	379.65
22	GA GS-3	1206781-66-9	-7.86	1,740
23	GA H	98665-19-1	-7.05	6,770
24	GA I	98665-20-4	-9.16	192.28
25	GA J	100440-26-4	-9.48	112.35
26	GA Ja	112430-67-8	-8.87	314.29
27	GA Jb	112430-68-9	-8.95	273.93
28	GA K	104700-95-0	-8.4	696.77
29	GA L	102607-24-9	-9.54	101.48
30	GA LM2	508182-41-0	-8.39	713.51
31	GA M	110311-47-2	-9.36	138.4
32	GA Ma	108026-89-7	-6.17	30,240
33	GA Mb	108026-90-0	-6.89	8,900
34	GA Mc	108026-91-1	-7.07	6,610
35	GA Md	108026-92-2	-6.53	16,330
36	GA Me	108026-93-3	-7.09	6,350
37	GA Mf	108026-94-4	-8.77	369.98
38	GA Mg	110042-11-0	-7.18	5,430
39	GA Mh	110024-17-4	-7.04	6,900
40	GA Mi	110024-16-3	-8.03	1,290
41	GA Mj	110024-15-2	-7.19	5,360
42	GA Mk	110024-14-1	-7.97	1,440
43	GA N	110241-19-5	-9.2	181.04
44	GA O	110241-21-9	-10.12	38.28
45	GA P	112667-14-8	-7.3	4,450
46	GA R	103963-39-9	-7.46	3,410
47	GA S	104759-35-5	-8.78	369.21
48	GA SZ	865543-37-9	-8.99	255.68
49	GA T	103992-91-2	-5.73	62,870
50	GA TN	112430-64-5	-7.67	2,400
51	GA TR	862893-75-2	-9.14	199.55
52	GA TR1	1225286-05-4	-9.32	148.21
53	GA T-Q	112430-66-7	-8.1	1,160
54	GA U	86377-51-7	-8	1,360
55	GA V	86377-50-6	-7.29	4,570
56	GA V1	150033-91-3	-8.55	542.99
57	GA W	86377-49-3	-6.75	11,270
58	GA X	86377-53-9	-7.97	1,440
59	3-β-hydroxy GA X	-	-7.86	1,730
60	GA Y	86377-52-8	-8.49	602.92
61	GA Z	86420-19-1	-8.18	1,020
62	GA α	220181-81-7	-7.65	2,460
63	GA β	217476-76-1	-8.46	631.78
64	GA γ	294674-00-3	-8.83	338.2
65	GA δ	294674-02-5	-8.96	272.38
66	GA ε	294674-05-8	-8.83	338.29
67	GA ζ	294674-09-2	-10.16	35.86
68	GA η	294674-12-7	-8.2	979.27
69	GA θ	294674-15-0	-8.77	370.79
70	Ganodermic acid S	112430-63-4	-7.7	2,290
71	Ganodermic acid T-O	112430-65-6	-8.59	507.25
72	Ganoderenic acid A	100665-40-5	-8.69	426.02
73	Ganoderenic acid A	-	-9.49	111.51
74	Ganoderenic acid B	100665-41-6	-6.89	8,860
75	Ganoderenic acid C	100665-42-7	-6.89	8,910
76	Ganoderenic acid D	100665-43-8	-7.17	5,590
77	Ganoderenic acid E	110241-23-1	-6.8	10,380
78	Ganoderenic acid F	120462-47-7	-7.63	2,550
79	Ganoderenic acid G	120481-73-4	-7.71	2,230
80	Ganoderenic acid H	120462-48-8	-7.54	2,970
81	Ganoderenic acid I	120462-49-9	-7.35	4,120
82	Ganoderenic acid K	942950-94-9	-7.72	2,180
83	Methyl GA A	81907-63-3	-9.1	214.48
84	Methyl GA AP	120462-52-4	-8.94	280.93
85	Methyl GA B	81907-65-5	-7.8	1,910
86	Methyl GA D	97210-12-3	-9.07	225.56
87	Methyl GA Df	1351348-00-9	-10.28	29.18
88	Methyl GA DM	-	-8.51	573.98
89	Methyl GA E	98718-43-5	-9.58	95.72
90	Methyl GA F	98665-08-8	-9.97	49.26
91	Methyl GA G	98665-23-7	-8.56	531.94
92	Methyl GA H	98665-11-3	-7.1	6,220
93	Methyl GA I	98683-73-9	-8.15	573.68
94	Methyl GA K	110414-79-4	-9.17	189.33
95	Methyl GA K2003	105742-77-6	-8.86	318.33
96	Methyl Ganoderenic acid H	120462-54-6	-7.25	4,860
97	Methyl Ganoderenic acid I	120462-53-5	-7.18	5,480
98	Ethyl GA F	1245946-63-7	-10.16	35.97
99	Propyl GA F	-	-10.27	29.47
100	i-Propyl GA F	-	-10.14	36.96
101	Butyl GA F	-	-10.09	40.43
102	i-Butyl GA F	-	-10.34	26.23
103	s-Butyl GA F	-	-9.27	75.21
104	t-Butyl GA F	-	-10.17	35.18
105	Butyl GA A	1207106-19-1	-9.41	126.76
106	Butyl GA B	1207106-20-4	-8.46	634.33
107	Tri-OAc Ganodermatriol	1028449-54-8	-9.21	176.11
108	Ganodermatriol M	-	-9.31	150.67
109	3-OAc GA B	-	-8.47	619.16
110	12-hydroxy GA C2	942936-52-9	-8.99	256.31
111	12-α-hydroxy GA D	-	-9.94	51.72
112	12-α-OAc GA D	942936-55-2	-9.84	61.51
113	15-OAc Ganolucidic acid E	1309931-94-9	-7.64	2,500
114	12-hydroxy GA F	-	-10.29	28.49
115	20-hydroxy GA G	400604-12-8	-9.76	70.07
116	3-OAc GA K	-	-8.79	358.12
117	3-OAc GA H	942936-56-3	-7.07	6,570
118	7-methyl GA O	112667-15-9	-7.3	4,490
119	7-ethyl GA O	-	-6.32	23,130
120	15-hydroxy GA S	-	-9.07	223.84
121	23-hydroxy GA S	1225286-07-6	-8.7	422.67
122	7-carbonyl GA Z	-	-8.36	742.06
123	7-carbonyl methyl GA Z	-	-7.83	1,820
124	2-OAc methyl GA A	81907-64-4	-7.53	3,030
125	7-hydroxy methyl GA AP	120481-75-6	-9.1	214.81
126	Me89GA J	400604-11-7	-9.65	84.48
127	Lucidenic acid A	95311-94-7	-8.31	807.65
128	Lucidenic acid B	95311-95-8	-8.2	981.84
129	Lucidenic acid C	95311-96-9	-7.51	3,130
130	Lucidenic acid D	98665-16-8	-9.1	212.66
131	Lucidenic acid D1	97653-95-7	-9.01	250.71
132	Lucidenic acid E	98665-17-9	-8.57	523.96
133	Lucidenic acid E1	97673-89-7	-8.24	911.36
134	Lucidenic acid F	98665-18-0	-9.05	231.93
135	Lucidenic acid G	102607-21-6	-8.09	1,180
136	Lucidenic acid H	110241-25-3	-7.71	2,230
137	Lucidenic acid I	110241-27-5	-9.54	101.02
138	Lucidenic acid J	110241-29-7	-8.7	422.78
139	Lucidenic acid K	110241-31-1	-8.86	318.4
140	Lucidenic acid L	110267-45-3	-8.46	633.21
141	Lucidenic acid M	110241-33-3	-8	1,370
142	Lucidenic acid O	250643-33-5	-7.49	3,250
143	Lucidenic acid P	648430-31-3	-7.32	4,310
144	Lucidenic acid SP1	364622-33-3	-7.77	2,030
145	20deHLA A	852396-69-7	-7.57	2,800
146	20deHLA N	1206781-67-0	-7.39	3,860
147	20OHLA A	1206781-68-1	-8.25	894.69
148	20OHLA D2	852936-71-1	-8.88	310.28
149	20OHLA E2	852567-75-0	-8.71	411.51
150	20OHLA F	852567-72-7	-9.16	192.27
151	20OHLA N	852567-78-3	-8.16	1,040
152	20OHLA P	852567-80-7	-7.74	2,130
153	LAlactone	250643-34-6	-6.91	8,640
154	Lucideraldehyde A	420781-84-6	-7.79	1,960
155	Lucideraldehyde B	480439-84-7	-8.41	687.6
156	Lucideraldehyde C	252351-96-5	-8.45	644.96
157	Lucideraldehyde D	873061-78-0	-8.29	837.72
158	Ganolucidic acid A	98665-21-5	-9.77	69.51
159	Ganolucidic acid B	98683-75-1	-9.42	123.9
160	Ganolucidic acid C	100440-27-5	-8.77	370.2
161	Ganolucidic acid D	102607-22-7	-9.16	193.85
162	Ganolucidic acid E	114567-50-9	-9.05	234.28
163	Ganosporic acid A	135357-25-4	-9.66	83.46
164	trideOAcGA_T	116763-90-7	-8.52	571.65
165	MeLA A	105742-79-8	-7.24	4,910
166	MeLA C	98094-88-3	-8.17	1,020
167	MeLA D2	98665-09-9	-8.88	311.87
168	MeLA E2	98665-12-4	-8.4	692.6
169	MeLA F	98665-10-2	-8.92	291.39
170	MeLA L	110267-46-4	-8.34	767.46
171	MeLA N	1276655-49-2	-7.62	2,620
172	MeLA P	647856-35-7	-7.11	6,120
173	MeLA Q	648430-32-4	-8.03	1,310
174	MeGlA A	98665-13-5	-9.48	111.8
175	MeGlA B	98683-74-0	-9.32	146.31
176	MeGlA D	102607-26-1	-9.04	234.95
177	Me20deHLA A	852936-70-0	-7.19	5,340
178	BuLA A	1207106-22-6	-8.36	740.68
179	BuLA B	1314143-37-7	-8.23	928.49
180	BuLA N	1207106-21-5	-7.35	4,090
181	Ganoderal A	106518-61-0	-7.74	2,130
182	Ganoderal B	106518-62-1	-9.56	97.8
183	Ganoderal F	114567-47-4	-9.17	190.81
184	Ganodermadiol	104700-96-1	-7.77	2,000
185	Ganodermatriol	105300-28-5	-8.06	1,230
186	Ganodermanonol	104700-97-2	-8.6	494.44
187	GAdiol 2006	107900-76-5	-8.66	452.08
188	GAtriol	106518-63-2	-8.47	622.13
189	89epoxyGA C	-	-9.08	221.51
190	89GA C	-	-8.73	401.72
191	89GA J	400604-10-6	-8.89	303.47
192	Ganolactone A	173268-82-1	-7.86	1,740
193	Ganolactone B	1028449-53-7	-7.72	2,210
194	Gsl A	138008-04-5	-8.44	650.14
195	Gsl B	138008-05-6	-7.37	3,960
196	Furano GA	120481-74-5	-7.12	6,060
197	EpGOH A	114020-56-3	-8.04	1,280
198	EpGOH B	114020-57-4	-8.36	739.99
199	EpGOH C	114020-58-5	-8.25	896.93
200	GaldTR	1225286-06-5	-8.75	385.99
201	GamdT	1341220-87-8	-7.25	4,860
202	Ganoderal A	104700-98-3	-8.4	694.04
203	Ganoderal B	114020-55-2	-7.76	2,070
204	Ganoderon B	252351-95-4	-7.42	3,620
205	Ganoderone A	873061-79-1	-8.26	882.3
206	Ganoderone C	873061-80-4	-8.47	615.75
207	Lucidumol A	217476-73-8	-9.08	222.02
208	Lucidumol B	107900-79-8	-8.8	356.42

Abbreviations: rG, free binding energy; Ki, inhibition constant; GA, ganoderic acid; pG4DNA, parallel G-quadruplex DNA; GLTs, triterpenoids isolated from Ganoderma lucidum.				

**Table 2:** MM/GBSA binding energies of GA A and GA Df to pG4DNA and residues involved in the G4-ligand interactions.

Triterpenoids	ΔVDWa (kcal/mol)	ΔSURb (kcal/mol)	ΔGBELEc (kcal/mol)	ΔTOTd (kcal/mol)	Residues involved in H-bonding	H-bond length (Å)
GA A	-49.47 ± 1.57	-3.93 ± 0.04	29.94 ± 1.61	-23.46 ± 1.70	DG 11	2.21
GA Df	-44.76 ± 2.17	-3.84 ± 0.04	35.28 ± 2.51	-13.32 ± 2.21	DG 11	2.12
					DA 3	2.74

a ΔVDW is the change in van der Waals energy in the gas phase upon complex formation. b ΔSUR is the change in energy due to the change in surface area upon complex formation. c ΔGBELE is the change in GB reaction field energy + gas phase electrostatic energy upon complex formation. d ΔTOT = ΔVDW + ΔSUR + ΔGBELE is the change in potential energy in water upon complex formation.						
Abbreviations: MM-GBSA, molecular mechanics/ generalized Born surface area; DA 3, adenine base of G-quadruplex DNA at position 3; DG 11, guanine base of G-quadruplex DNA at position 11; GA, ganoderic acid.
